# Retraction Note: Enhancing MPPT performance for partially shaded photovoltaic arrays through backstepping control with Genetic Algorithm-optimized gains

**DOI:** 10.1038/s41598-025-86995-9

**Published:** 2025-01-21

**Authors:** Serge Raoul Dzonde Naoussi, Kenfack Tsobze Saatong, Reagan Jean Jacques Molu, Wulfran Fendzi Mbasso, Mohit Bajaj, Mohamed Louzazni, Milkias Berhanu, Salah Kamel

**Affiliations:** 1 Technology and Applied Sciences Laboratory, U.I.T. of Douala, University of Douala, P.O. Box 8689, Douala, Cameroon; 2 Unité de Recherche d’Automatique et d’Informatique Appliquée, I.U.T. Fotso Victor, University of Dschang, P.O. Box 134, Bandjoun, Cameroon; 3 Department of Electrical Engineering, Graphic Era (Deemed to Be University), Dehra Dun, 248002 India; 4 Hourani Center for Applied Scientific Research, Al-Ahliyya Amman University, Amman, Jordan; 5 Graphic Era Hill University, Dehra Dun, 248002 India; 6 Applied Science Research Center, Applied Science Private University, Amman, 11937 Jordan; 7 Science Engineer Laboratory for Energy LabSIPE, National School of Applied Sciences ENSAJ, Chouaib Doukkali University, 24000 El Jadida, Morocco; 8 Department of Electrical and Computer Engineering, Addis Ababa Science and Technology University, Adama, Ethiopia; 9 Department of Electrical Engineering, Faculty of Engineering, Aswan University, Aswân, 81542 Egypt

Retraction of: *Scientific Reports* 10.1038/s41598-024-53721-w, published online 09 February 2024

The Editors have retracted this Article.

After publication, concerns about similarities between this Article and a separate publication with no common authors^1^ were brought to the attention of the Editors. Specifically, Figures 1 and 4-7 and Tables 1 and 2 are all similar to material published in that article, and there are also significant textual similarities between the two publications.

Serge Raoul Dzonde Naoussi, Reagan Jean Jacques Molu, Wulfran Fendzi Mbasso, Mohit Bajaj, Milkias Berhanu, and Salah Kamel agree with this retraction. Kenfack Tsobze Saatong and Mohamed Louzazni did not respond to the correspondence from the Editors about this retraction.
